# Challenges and Opportunities in Developing an Oncology Clinical Trial Network in the United States Veterans Affairs Health Care System: The VA STARPORT Experience

**DOI:** 10.3390/curroncol31080358

**Published:** 2024-08-21

**Authors:** Abhishek A. Solanki, Kevin Zheng, Alicia N. Skipworth, Lisa M. Robin, Ryan F. Leparski, Elizabeth Henry, Matthew Rettig, Joseph K. Salama, Timothy Ritter, Jeffrey Jones, Marcus Quek, Michael Chang, Alec M. Block, James S. Welsh, Aryavarta Kumar, Hann-Hsiang Chao, Albert C. Chen, Ronald Shapiro, Rhonda L. Bitting, Robert Kwon, William Stross, Lindsay Puckett, Yu-Ning Wong, Nicholas G. Nickols, Kimberly Carlson

**Affiliations:** 1 Edward Hines Jr. VA Hospital, Hines, IL 60141, USAalec.block@va.gov (A.M.B.);; 2 Department of Radiation Oncology, Stritch School of Medicine, Loyola University Chicago, Maywood, IL 60153, USA; 3 Cooperative Studies Program, VA Connecticut Healthcare System, West Haven, CT 06515, USA; 4 Cooperative Studies Program, Edward Hines Jr. VA Hospital, Hines, IL 60141, USAkimberly.carlson@va.gov (K.C.); 5 VA Greater Los Angeles Healthcare System, Los Angeles, CA 90073, USA; matthew.rettig@va.gov (M.R.);; 6 Durham VA Medical Center, Durham, NC 27705, USA; 7 Richmond VA Medical Center, Richmond, VA 23249, USA; 8 Michael E. DeBakey VA Medical Center, Houston, TX 77030, USA; jeffrey.jones9@va.gov (J.J.);; 9 Department of Urology, Stritch School of Medicine, Loyola University Chicago, Maywood, IL 60153, USA; 10 Louis Stokes Cleveland VA Medical Center, Cleveland, OH 44106, USA; 11 Richard L. Roudebush VA Medical Center, Indianapolis, IN 46202, USA; 12 VA Long Beach Healthcare System, Long Beach, CA 90822, USA; 13 Minneapolis VA Medical Center, Minneapolis, MN 55417, USA; 14 Clement J. Zablocki VA Medical Center, Milwaukee, WI 53295, USA; lindsay.puckett@va.gov; 15 Corporal Michael J. Crescenz VA Medical Center, Philadelphia, PA 19104, USA

**Keywords:** prostate cancer, randomized clinical trial, oligometastasis, recruitment, radiotherapy, veterans, site selection

## Abstract

The United States Veterans Affairs (VA) Health Care System has a strong history of conducting impactful oncology randomized clinical trials (RCTs). We developed a phase II/III RCT to test the use of metastasis-directed therapy in Veterans with oligometastatic prostate cancer (OMPC)—the first VA RCT in OMPC that leverages novel imaging and advanced radiotherapy techniques. To accomplish this, we developed a clinical trial network to conduct the study. In this manuscript, we describe several challenges we encountered in study development/conduct and our strategies to address them, with the goal of helping investigators establish robust study networks to conduct clinical trials. In the study start-up, we encountered challenges in timely site activation, and leveraged project management to maximize efficiency. Additionally, there were several changes in the clinical paradigms in imaging and treatment that led to protocol amendments to ensure maximum equipoise, recruitment, and impact of the study. Specifically, we amended the trial to add de novo OMPC patients (from initially only recurrent OMPC) and expanded the study to allow up to 10 metastases (from initially five). Finally, in order to maintain local study team engagement, we developed initiatives to maximize collaboration and add value to the overall clinical program through study participation.

## 1. Introduction

The United States Veterans Affairs (VA) Health Care System has a long history of conducting landmark trials in oncology. The VA Larynx trial demonstrated that patients with locoregionally advanced laryngeal cancer could have organ preservation through chemotherapy and radiation [1], and the PIVOT trial demonstrated that many men with localized prostate cancer could safely avoid prostatectomy without compromising survival [2]. The results of these trials have established and strengthened clinical standards of care and been incorporated into the VA and national guidelines [3,4].

There are several attributes that make the VA a robust environment to conduct oncology clinical trials. The presence of an integrated electronic health record (EHR) system and embedded research infrastructure allows for the ease of data collection and coordination, both centrally and locally. Moreover, given the unique Veteran patient population, there is a culture of altruism and service that is uniquely present and facilitates participation in research. For instance, a recent survey of Veterans receiving care at the Bronx VA found that 78.5% of Veterans trusted doctors who do medical research, and 87.5% stated they would strongly consider joining a trial if their VA primary care physician recommended it [5]. Importantly, 93.8% responded that they would participate in a clinical trial if it would help fellow Veterans in the future [5]. Additionally, unlike patients covered by commercial insurance or Medicare, Veterans pay much lower copayments for their oral anticancer medications, reducing the financial barriers to care observed in other health care settings. Also, Veterans can schedule appointments with providers in other specialties, including radiology, without referrals or prior authorizations needed. Furthermore, several cancers are particularly prevalent at a higher rate than in the civilian population, including prostate, lung, and head and neck cancers [6]. Finally, randomized trials that compare intervention and nonintervention (e.g., watchful waiting vs. radical prostatectomy for early-stage prostate cancer) are challenging to conduct for many reasons. Unlike civilian medical centers, the absence of a fee-for-service remuneration model uniquely facilitates more balanced and open discussions about the pros/cons of participation in a randomized clinical trial.

Metastatic prostate cancer is a particularly challenging disease entity with rapidly evolving multimodality treatment approaches and highly sensitive diagnostic tests and imaging. Historically, patients with metastatic prostate cancer were considered to have incurable, widespread disease, and systemic therapy was thought to be the only meaningful treatment option. Local therapy was therefore reserved primarily for palliation, as the treatment paradigm suggested that aggressive local therapy would be futile since distant sites would rapidly progress. This would lead to patients suffering the toxicities of aggressive local therapy without any meaningful benefit. However, increasing clinical data demonstrate that a large proportion of patients have “oligometastatic” prostate cancer, which is defined as metastatic prostate cancer that involves only a limited number of anatomic sites with a limited number of lesions. It has been hypothesized that aggressive local therapy to the oligometastatic lesions using radiation or surgery can lead to durable cancer control and potentially cure in some [7]. Several phase II trials have demonstrated a signal of efficacy of metastasis-directed therapy (MDT) [8,9,10,11]. However, none of the trials was designed to be definitive. The patients who were enrolled in these studies were determined to have oligometastasis based primarily on conventional and older molecular imaging approaches. PSMA PET/CT has greater sensitivity for identifying nodal and bone metastases and was rapidly integrated into practice in both the VA and civilian centers [12,13,14]. Thus, oligometastasis is more effectively being detected earlier in the disease course, even before the onset of symptoms. Furthermore, in most of these studies, the control arm was primarily surveillance. Multiple, definitive, phase III randomized trials have demonstrated the best survival for recurrent or de novo metastatic prostate cancer is achieved using earlier, enhanced systemic therapy that combines androgen deprivation therapy (ADT) with androgen receptor pathway inhibitors (ARPI) or chemotherapy, and is considered today’s standard systemic therapy (SST) [15].

It is in this context that we developed the Veterans Affairs seamless phase II/III randomized trial of **STA**ndard systemic the**R**apy with or without **P**ET-directed local therapy for **O**ligometastatic p**R**os**T**ate cancer (VA STARPORT; NCT04787744). We aimed to utilize the strengths of the VA oncology clinical trial environment to definitively determine the role of MDT in the setting of contemporary systemic therapy and PSMA PET/CT patient selection to maximize the impact of the study. VA STARPORT is a phase II/III randomized trial comparing SST and SST with PET-directed local therapy using radiation or surgery. It is the first randomized trial in the VA to utilize novel prostate cancer PET/CT imaging and advanced radiotherapy methods. Given the rapid evolution of imaging practices, MDT approaches, and SST practices, our guiding principle was to create a trial network that would maximize local study team engagement to facilitate recruitment and allow the study to adapt to changes in clinical practices to maximize the long-term impact of the study - all without compromising internal or external validity. In this manuscript, we discuss our strategies for designing VA STARPORT, challenges encountered during the study conduct, and the strategies to address them, with the goal of creating a resource to help other investigators successfully develop multisite clinical trials.

## 2. Materials and Methods

### 2.1. Study Design

From the conception of VA STARPORT, our goal was to develop a study that utilized multidisciplinary leadership and “grass roots” development to develop a scientifically robust, yet practical and generalizable study. The study was developed as a collaboration between the VA Clinical Science Research and Development (CSRD) program (the funding agency), and the VA Cooperative Studies Program (CSP; a clinical research infrastructure specializing in the design and oversight of large-scale clinical trials and epidemiological studies by providing methodological, technical, and administrative support). CSP has a long history of conducting collaborative comparative effectiveness research that has influenced clinical care [16,17].

**Figure 1:**
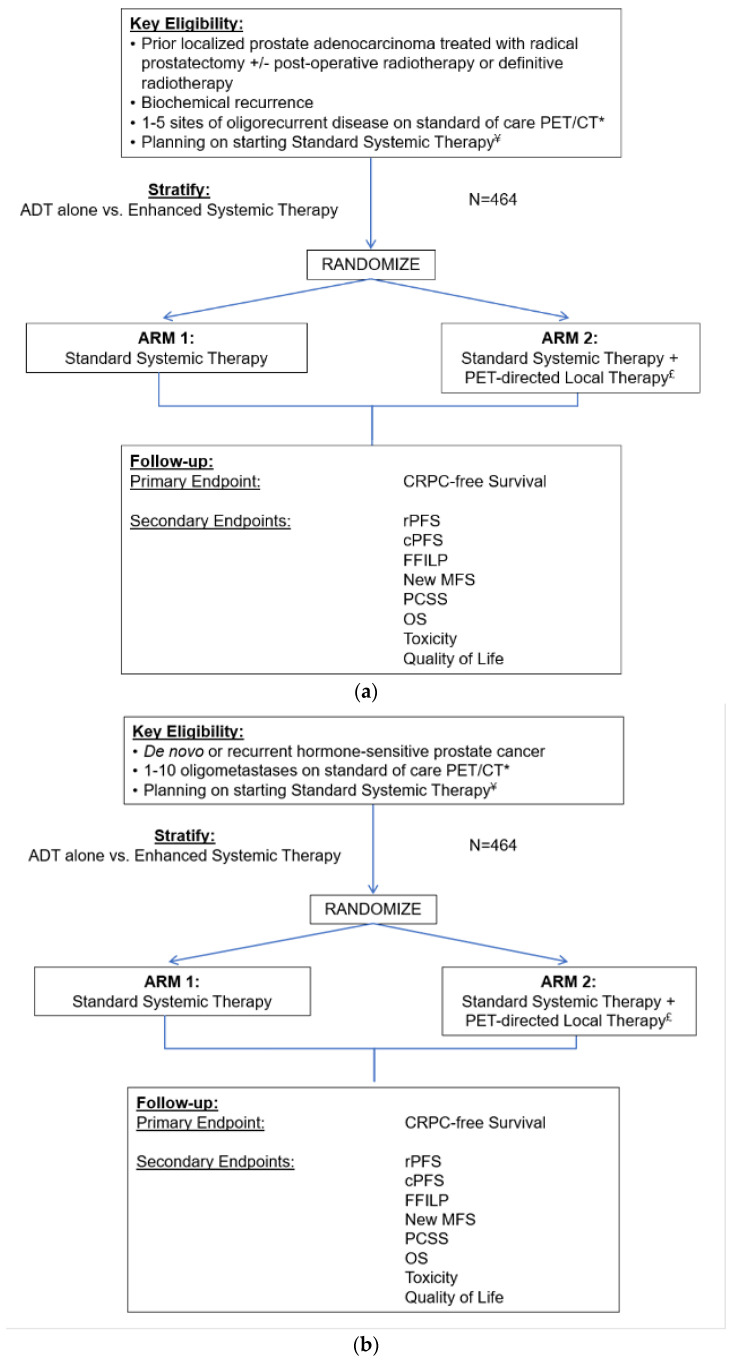
The original VA STARPORT study schema (**a**) and the current amended STARPORT study schema that includes de novo patients and up to 10 metastases (**b**). ADT = androgen deprivation therapy; CRPC = castration-resistant prostate cancer; rPFS = radiographic progression-free survival; cPFS = clinical progression-free survival; FFILP = freedom from local progression; MFS = metastasis-free survival; PCSS = prostate cancer-specific survival; OS = overall survival. * Includes any form of molecular PET/CT imaging for prostate cancer. ¥ Standard systemic therapy will be determined by the treating physician and must be consistent with current NCCN guidelines. It can be delivered by using: (1.) ADT or (2.) Enhanced system therapy delivered by combining ADT with any chemotherapy or androgen receptor axis targeted agent. £ Metastasis-directed therapy can be delivered with surgery or radiation, and those with local recurrence will be treated with local therapy per standard of care.

Together with the CSP team, a study team consisting of medical oncologists, radiation oncologists, and urologists at several VA medical centers that reflected the diversity of institution size and geography, in collaboration with non-VA external advisors, developed the initial study design. Figure 1a depicts the original study schema. The study initially enrolled Veterans with recurrent oligometastatic disease, with 1–5 metastases on PET/CT imaging, and randomizes between SST alone (Arm 1) or with PET-directed local therapy using surgery or radiation to the metastases and any local recurrence (Arm 2). Figure 1b depicts the current amended study schema that includes the de novo and 6 to 10 metastases populations.

### 2.2. Site Selection

For feasibility approximation, we used the VA central repository of Veteran health data (Corporate Data Warehouse) using the VA Informatics and Computing Infrastructure (VINCI), an initiative to allow researchers better access to VA data. Estimates from VINCI allowed for the approximation of the number of Veterans with prostate cancer who had recurrence after definitive radiation or surgery and had a biochemical recurrence and PET/CT scan. This allowed us to identify the centers with the largest potential participant pool. Additional selection criteria included on-site medical oncology, radiation oncology, and urology services. Potential sites were sent a site survey, which included questions on equipoise, radiation and surgery treatment capabilities, availability of PSMA PET/CT technology, and research infrastructure. The site survey is attached as a Supplemental Document.

We ranked all the VA medical centers based on the number of potential participants in this analysis and then applied the below selection criteria, trying to choose sites higher on the list. We selected sites with on-site radiation oncology and the ability to deliver SBRT and surgery for MDT. From this list, we preferentially selected sites that participated in the **V**eterans **A**ffairs **L**ung cancer surgery **O**r stereotactic **R**adiotherapy (VALOR) trial of lobectomy or stereotactic radiation in lung cancer (NCT02984761), as these sites had expertise in clinical trial accrual and conduct, as well as the required radiation QA infrastructure to participate in a trial of this nature. Local site investigators (LSIs) were identified from the selected sites based on expertise in treating oligometastases, expertise in oligometastasis clinical trial design, as well as experience as an investigator in a large clinical trial. Priority was aimed towards sites with scientific and clinical expertise in prostate cancer, such as selection as a VA/PCF (Prostate Cancer Foundation, Santa Monica, CA, USA) center of excellence. We then performed a site performance and feasibility survey of all interested sites. Our site selection survey was designed using CSP methodologies and asked details regarding trial feasibility and site capabilities. There were several key findings: (1) All sites had enthusiasm and equipoise for this study question. (2) All sites could deliver the MDT and salvage local therapy needed for our study. (3) All sites had the technical and personnel resources to successfully enroll and follow patients, as well as coordinate the translational endpoints. (4) There were no competing trials for these patient populations at these sites. (5) Sites expressed capacity (as based on potentially eligible participants) of enrolling 1 to 3 participants per month.

### 2.3. Protocol Development

During protocol development, every aspect of the protocol, including patient selection, interventions, and follow-up assessments was shared with the local site investigators for feedback, and suggestions were incorporated into the final version of the protocol. Metastasis-directed radiotherapy (MDRT) is a particularly complex treatment approach with several acceptable standards of care and is a part of the study. We conducted a survey of sites as part of the RT credentialing process to determine the favored approach (stereotactic body radiotherapy vs. elective nodal radiotherapy with a simultaneous integrated boost) [18]. Ultimately, we determined the heterogeneity of the patient population being enrolled in the study, and the heterogeneity of the opinion of the VA radiation oncologists regarding the optimal RT approach, necessitated allowing both strategies, with the goal of preventing the type of radiation delivered on protocol from being a barrier to enrollment.

Given our integrated VA EHR and the availability of telehealth technology, we incorporated decentralized elements, such as central data collection through limited participation for participants who could not continue protocolized follow-ups due to relocation or other reasons. Access to robust telehealth resources and a relatively “closed” medical system of the VA in which Veterans, particularly those who participate in VA STARPORT, receive care, both allowed for robust data collection and minimal missing data.

## 3. Results

Study development began in October 2020 with a plan to include 16 VA medical centers. The study opened in July 2021. Table 1 depicts the several challenges encountered, and the strategies used to address them. These are discussed in detail below.

### 3.1. Challenge #1: Developing a Clinical Trial Network

**Figure 2:**
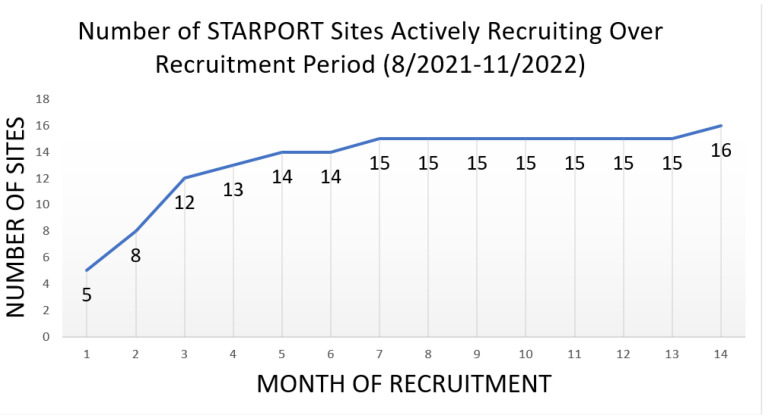
Sites enrolling in VA STARPORT over the first phase of recruitment until all sites became active.

There were several challenges that the central and local study teams encountered immediately upon initiating the study. Only five sites were able to initiate enrollment in the first month following the study kick-off. Five other sites did not have a study coordinator available at the time of the study start-up, which led to a delay in approval to begin recruitment. An additional three sites opened for enrollment in August 2021. The remaining initial sites opened from September 2021 to January 2022 due to hiring issues and regulatory approval delays. Finally, the study opened in the depths of the COVID-19 pandemic, leading to delays. Figure 2 depicts the number of sites enrolling patients in STARPORT over the first phase of recruitment until all sites became active. Note that from the study kick-off, it took 14 months for all of the 16 original sites to become active. Table 2 describes several key study start-up milestones. Of note, it took sites an average of 10.1 months (median: 9.7 months) to hire a study coordinator after they were officially selected for the trial (PMO or Merit Review Approval). Once a site was open to enrollment, it took an average of 3.3 months (median: 2.6 months) for a site to enroll their first participant.

Our strategies for facilitating rapid site activation included an all-staff training session for protocol and operation procedures, supporting sites to fulfill regulatory requirements, providing sites a general position description for hiring study coordinators, monitoring start-up trends on site readiness and activities, and collecting and storing investigator qualification documents. As sites began recruitment, the central study team developed and continues to manage the study timeline to monitor individual site recruitment, as well as overall study progress. The study team also maintains the clinical trial management system (CTMS) both for maintaining required regulatory documentation and to facilitate centralized team communication.

Another challenge during the initial phases of the study was the limited research experience of the study teams at some of the local sites that were newer to clinical trial research. To establish best practices for both recruitment and research safety at each of the sites, we initiated virtual site visits early and frequently at each of the sites. In these meetings, we discussed the unique recruitment pathway of participants at each site and provided recruitment tools. We also attempted to identify workflow or patient interaction barriers to recruitment to address them early on. Through these discussions, we learned that the key steps for successful recruitment were as follows: (1.) ensuring that PSMA PET/CT imaging was performed routinely for the eligible patient population, (2.) the local research team had access to the results of the PET/CT imaging results, and (3.) the study team reached out early to the ordering clinician in order to facilitate a unified approach in discussing the clinical trial and management options. Importantly, most study sites are teaching sites for their academic affiliate institutions, allowing residents and fellows to frequently participate in clinical care. We learned that conflicting messages from trainees and the other members of the clinical team who may have less experience with the clinical trial were a major risk to recruitment. To address this, we developed a “script” of example dialogue to facilitate equipoise in discussions with potential participants.

### 3.2. Challenge #2: Adapting to the Shifting Sands of Clinical Practice in Metastatic Prostate Cancer

While rapid advances in diagnostics and treatments for patients with cancer is unquestionably a positive, it can make the conduct of a large scale clinical trial challenging, and can limit the final impact of the study if practices change drastically during the course of the trial. Keeping on pace with these changes in eligibility, diagnostics, and interventions in VA STARPORT to reflect the standard of care has been a challenge. For example, VA STARPORT was developed in the era of 18F-Fluciclovine PET/CT as the only available PET/CT imaging modality for prostate cancer. Moreover, it was specifically FDA-approved for biochemically recurrent prostate cancer. Therefore, we initially designed VA STARPORT for recurrent oligometastatic patients only. However, within months of the initiation of recruitment, 18F-DCFPyL PSMA PET/CT and Ga68-PSMA-11 PET/CT were FDA-approved for both de novo and recurrent prostate cancer. This had two key implications for the study sites and STARPORT enrollment:

First, 18F-Fluciclovine PET/CT has less sensitivity than PSMA PET/CT and, therefore, conventional imaging with CT and bone scans were still required (and consequently built into the protocol). However, because PSMA PET/CT performs so well, conventional imaging was rarely used once PSMA PET/CT was made available at our VA facilities. As a result, requiring conventional imaging would have required participants to undergo unnecessary diagnostic procedures.. Consequently, the protocol was modified to make conventional imaging optional for the PSMA PET/CT-staged patients.

Second, the timing of PET/CT imaging, and consequently the timing of the diagnosis of oligometastatic prostate cancer, shifted from the recurrent setting to primarily in the de novo setting across our sites. The result was an entirely new disease category that became a major part of the clinicians’ practice in the VA, and one without a standard of care—similar to recurrent oligometastatic prostate cancer. Importantly, based on the initial study eligibility of recurrence after the initial local therapy for localized cancer, this patient population would never be eligible for VA STARPORT due to metastatic disease at diagnosis. Exploratory discussions demonstrated that the local study teams had equipoise and enthusiasm for enrolling the de novo patient population in VA STARPORT. Around this time, the results of the de novo and recurrent oligometastasis EXTEND trial were reported. This was a phase II randomized trial of 87 patients with prostate cancer that compared intermittent SST with or without MDT. EXTEND included recurrent (72%) and de novo (28%) patients with up to five metastases. This revealed a signal of a progression-free survival benefit of MDT. Importantly, subgroup analysis revealed a similar hazard ratio between those with recurrent and de novo disease, providing the most robust data to allow for the inclusion of de novo patients in VA STARPORT without a statistical redesign. Consequently, VA STARPORT was amended in January 2024 to allow for recurrent and de novo oligometastasis.

In parallel to these changes, the definition and practice patterns of oligometastasis have been rapidly evolving since the initiation of VA STARPORT. Upon discussion with our local study teams, new prostate cancer data, including that from the EXTEND trial [11], led to challenges with the clinicians’ equipoise of restricting enrollment to ≤5 metastases in the recurrent setting. The central study team reviewed the data and we consequently determined that these results would hinder enrollment and decrease the impact of VA STARPORT’s final results. In order to address this issue and maximize the innovation and impact of VA STARPORT, the study was amended in January 2024 to increase the number of eligible metastases to 10. This was based on the SABR-COMET-10 trial [19], an ongoing study that allowed up to 10 sites of metastasis. Additionally, consensus guidelines argue that there is no biologic rationale for the definition of oligometastasis as ≤5 metastases, and thus the use of five metastases as the top limit was arbitrary [20]. In fact, there are no strong data showing that in the PSMA PET/CT-defined metastatic setting there is a difference between 1–5 and 6–10 metastases. Figure 1b depicts the amended study schema.

From a trial design perspective, it is crucial to note that adapting to these trends required the largest and most complex amendment of the study. Adding participants with de novo prostate cancer and 6–10 metastases required considerable updates to the electronic data capture system, and the size of the amendment required constant coordination and collaboration among central and local study teams. The combination of ideation, database implementation, and regulatory approvals took over one year to implement. However, our study team felt that, while amendments of this size can be arduous, they are well worth the effort when fueled by ground-breaking changes in clinical practice. While VA STARPORT was enrolling at 43% of the expected randomizations before the amendment, the study has been enrolling at 58% of the expected randomizations in the first 3 months since the amendment, which is a 35% increase.

### 3.3. Challenge #3: Maintaining Local Study Team Engagement over the Course of the Study

Despite the enthusiasm of the coordinating center and the national PI, maintaining local site engagement and enthusiasm for a multiyear clinical trial can be challenging. The central study team attempted several interventions to add value to the local clinical and research programs in effort to maximize local site engagement. We attempted to keep all stakeholders at the local sites integrated in the decisions on study conduct. It was critical to ensure that all specialties involved in the trial were engaged in these discussions. For example, questions about SST regimens were discussed with the local medical oncologists, and salvage re-irradiation approaches and MDRT approaches were developed through input from all of the local radiation oncologists. Importantly, engaging all of the specialists involved in this patient population also identified unexpected causes of recruitment challenges, differences in opinions related to equipoise, and also allowed to identify study champions, who we found could be from any specialty involved. These strategies facilitated continued engagement, ongoing investment, and making sure all input was welcomed and valued. Another method used for engagment was to invite speakers to lead discussions on various topics impacting VA STARPORT and the care of Veterans with prostate cancer. Examples of this include scientific experts to discuss advances in the clinical management of metastatic prostate cancer, advances in MDT, and challenges and best practices in PSMA PET/CT interpretation. Other examples include speakers discussing the perspectives of Veterans living with prostate cancer, patient advocacy, and increasing the enrollment of diverse participants. As a consequence of these efforts and the engagement of our sites, 48% of the participants enrolled in VA STARPORT are African American or Hispanic/Latino—a significantly higher proportion than other international prostate cancer clinical trials.

**Figure 3:**
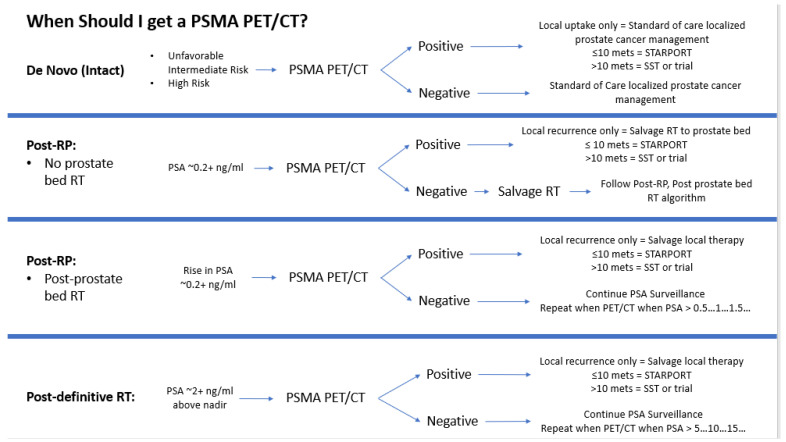
Algorithms for indications for PSMA PET/CT imaging and pathways for management.

The study team attempted to add value to the clinical programs through their participation in VA STARPORT to improve the care of Veterans at all sites. For example, we developed custom algorithms for the indications for PSMA PET/CT imaging in de novo and recurrent prostate cancerand pathways for management (Figure 3) that sites could modify to their institutional practices and post for other clinical colleagues who may not be as well-versed in prostate cancer management. Furthermore, through RT credentialing, we identified several opportunities at sites for improving the technical delivery of RT for both VA STARPORT and non-trial clinical practice.

We also hold monthly LSI meetings in which all investigators join to discuss the ongoing conduct of VA STARPORT, strategies for recruitment, as well as any central or local barriers and challenges. Importantly, we also include discussions regarding the latest data presented at conferences and publications on the management of metastatic prostate cancer to allow for the dissemination of best practices for both study and nonstudy patients. We discuss challenging clinical and RT planning situations, and strategies to deliver the optimal plan in difficult scenarios. Again, these learnings could be applied to both VA STARPORT and non-STARPORT patients. We also discuss other ongoing clinical trials to gain insights and efficiencies on similar research, and to identify other innovative studies that could be valuable additions to our local research programs. Finally, through the LSI meetings, LSIs are able to connect with other providers across the country to discuss their own research, helping to build camaraderie across VA STARPORT network.

We hold study coordinator group meetings monthly and 1:1 study coordinator meetings with the national coordinator twice a month. These meetings are particularly valuable as the group discusses site recruitment methods and pathways, Good Clinical Practice (GCP) guidelines, and site-specific challenges and strategies for the optimal conduct of study procedures. These forums allow for unique challenges to be addressed and the ability to expand on issues that are site specific in a protected, private environment. The meetings are also used to offer peer support for study coordinators and the opportunity to review the protocol to ensure the interpretation and applicaton were comprehensive and uniform. This was especially useful when bringing on new study coordinators. Periodic speakers—such as representatives from quality assurance, data management, and project management—are invited to the group meetings for onging guidance, oversight, and study-specific understanding. We also conduct individual virtual site visits between the national PI and national coordinator centrally, and the LSIs and study coordinators locally. These are conducted 1–2 times per year, and cover the above topics, but expand on both clinical considerations and research procedural considerations to help trouble shoot institutional-level issues. Frequently, lessons learned through one site’s resolution of issues can be disseminated to other sites through this mechanism, thereby helping sites overcome both clinical and research barriers through this collaboration. For example, several sites lost the ability to conduct PSMA PET/CT diagnostic studies on-site over the recruitment period. Strategies used to resolve the issue at one site were used by other sites to more rapidly regain access to PSMA PET/CT imaging.

One final strategy that has been helpful in understanding local site challenges and opportunities is that the leadership model for VA STARPORT is unique amongst other CSP studies in that the national PI (AAS) is also an LSI. This has allowed for the central study team to have a more granular view of the “front line” experiences of the study sites. It also has strengthed the relationship between the other LSIs and study leadership due to the shared experiences.

Study sites have also benefited from their participation in VA STARPORT. In a survey of the LSIs, 85% reported that the trial has helped standardize the management of biochemically recurrent, oligorecurrent, and de novo oligometastatic prostate cancer patients; 77% reported that participating in VA STARPORT helped improve the technical aspects of the clinical radiotherapy program; 62% reported that participating in VA STARPORT has allowed for the improved use of somatic and germline sequencing; and 43% reported participating in VA STARPORT helped them obtain access to PSMA PET/CT imaging quicker or more easily.

## 4. Discussion

In this manuscript, we discuss our endeavor to develop a clinical trial that leverages the strengths of the VA to conduct multisite oncology clinical trials to help address the challenges we encountered and the strategies we employed to navigate these challenges. Our manuscript is intended to be a resource for investigators who are developing clinical trials across clinical networks to provide a strategic roadmap for success. We found that close collaboration between the central study team and LSIs led to mutual benefit, and local sites were able to augment their clinical programs through participation in the clinical trial. In addition, VA STARPORT study protocol was modified to maximize feasibility at the sites and overall generalizability of the methods and strategies.

Randomized clinical trials are the gold standard for evidence generation regarding the comparative effectiveness of new therapeutic approaches in medicine, yet over half of oncology clinical trials are closed prematurely due to insufficient accrual [21]. The premature closure of a trial is a particularly negative outcome for funding agencies, patients, and the medical community, as it is a loss of not only time and financial investment, but also of a potential avenue for advances in care. Frequently cited challenges are inadequate research staff and resources, lack of awareness, bias regarding patient eligibility, preference for certain treatment, and insufficient time. For patients, issues include burdensome time demands, preferences for certain treatment, lack of awareness, stringent eligibility criteria, clinician influence, concerns about randomization, and logistical constraints [21].

The central study team encountered many of these challenges while running VA STARPORT. Our primary strategy for addressing these challenges was to strengthen the relationship between the central study team and the local study teams. This was accomplished through the immersive facilitation of local regulatory efforts and the hiring of research staff, adding value to the clinical program through participation in the study, and maximizing communication and collaboration with the study teams through regularly held meetings with local study teams facilitated by the central study team.

There have been multiple reports from prior clinical trials that have described aggregate conclusions regarding lessons learned from conducting clinical trials. Examples of this include the logical progression from preclinical to clinical trials in oncology, challenges from incorporating precision medicine, and insights into challenges in equipoise in oncology trials [22,23,24]. However, there are limited publications that describe strategies for developing clinical trial networks and engaging local sites to maximize study success. Our study provides strategic approaches that investigators can use when facing common challenges in clinical trial development and conduct.

Limitations of this study include that VA STARPORT is still an actively recruiting trial, and therefore the long-term scientific outcomes of the study, as well as the timing and completion of accrual, are unknown. Critically, VA STARPORT is only open in the VA Health Care System, and it is possible that some of our challenges and interventions are specific to this network. However, most of these challenges and strategies are likely generalizable to many health care environments. Unique attributes of the metastatic prostate cancer patient population may have influenced the types of challenges we encountered and the likelihood of success of our strategies for addressing them. We recognize that our strategies may be less effective in other health care settings, including malignant and nonmalignant disease states.

## 5. Conclusions

Planning, launching, and maintaining a clinical trial is a challenging undertaking, but VA STARPORT’s experience in its VA-centered environment provides a unique perspective on the process. The timetable for various study start-up events is a beneficial resource for studies in planning, and strategies for developing a clinical trial network, maintaining local site engagement, and adapting to shifting clinical practices are widely applicable. While VA STARPORT is still ongoing, the study’s experiences thus far can be a valuable reference for study teams and investigators when developing and conducting their own clinical trials.

## Figures and Tables

**Table 1:** Main challenges encountered in VA STARPORT and the respective strategies enacted to improve study performance and mitigate risk.

VA STARPORT: Summary of Main Challenges and Strategies to Address Them	
*Challenges*	*Strategies*
**Developing a clinical trial network** Delays in site initiation of enrollment. Difficulty hiring new coordinators. Extended timeline for regulatory approvals. Limited clinical trial research experience at some sites.	**Facilitating local success through central support** Support sites to fulfill regulatory requirements. Assist with hiring staff and maintaining investigator qualifications. Management of the Clinical Trial Management System. Facilitating regular review of study procedures, study protocol, and changes implemented by the VA Central Office. Early and frequent virtual site visits at each site to: Discuss recruitment pathways. Provide recruitment tools. Identify workflow or patient interaction barriers to recruitment. Provide a “script” to help discuss trial to facilitate equipoise.
**Shifts in clinical practice for prostate cancer treatment** Evolving approvals of PSMA PET/CT imaging in recurrent and de novo prostate cancer, and consequent shifts in imaging practices. Newly emerging clinical trial data in oligometastasis. Changing standard systemic therapy practices.	**Adapting the study protocol to evolving research** Maintaining awareness on the developments in prostate cancer research and trial results, and regular dialogue with investigators. Study responds to the current research environment through amending the study protocol. Hold study- and site-level trainings on changes made to the study protocol and any operational procedures. Allow for the incorporation of evolving standard of care approaches while continuously reassessing scientific impact to maximize practicality, generalizability, and impact of study.
**Maintaining local study team** **engagement** Faltering study coordinator attendance in all staff meetings. Sites’ limited understanding of study procedures. Lowering motivation over the length of the study.	**Maximize local site participation in study procedure development and provide value to local clinical programs through participation in the study** Local clinicians involved in the development of study treatments and assessments. Offer frequent and individualized study procedure trainings as local research staff turnover. Creation of custom clinical algorithms that sites can adapt to improve clinical pathways. Regular discussion with investigators on challenging clinical and RT scenarios, and strategies for implementing solutions. Scientific expert presentations at monthly all-staff meetings that are relevant to both study and non-study clinical care.

**Table 2:** Time frames of key milestone study start-up events of VA STARPORT participation.

	Time (Months)				
	Site Selection to Site Coordinator Hire	Site Selection to Local R&D Approval	Site Selection to Enrollment Start	Site Selection to First Consent	Enrollment Start to First Consent
Median	9.7	8.8	11	13.6	2.6
25th percentile	7.7	6.7	10.6	11.5	0.8
75th percentile	10.9	10.3	12.2	16.1	4.7
Average	10.1	8.7	10.8	14.1	3.3
Std Deviation	5.2	2.6	2.3	3.9	2.9
Minimum	1.3	4.3	5.1	7.8	0.2
Maximum	23.7	12.6	14.5	22.4	9.8

## Data Availability

No new data were created or analyzed in this study. Data sharing is not applicable to this article.

## Supplementary Materials

The following supporting information can be downloaded at: https://www.mdpi.com/article/10.3390/curroncol31080358/s1, VA STARPORT Site Selection Survey.
