# Supplementary material for: Challenges and Opportunities in Developing an Oncology Clinical Trial Network in the United States Veterans Affairs Health Care System: The VA STARPORT Experience
Source: Curr Oncol. 2024 Aug 21;31(8):4781–94. doi: 10.3390/curroncol31080358 (PMC11353739; doi:10.3390/curroncol31080358)
Supplement: Supplementary file 1 [file curroncol-31-00358-s001.zip › curroncol-3083572-supplementary.pdf]

We are writing to update you on the progress of the VA STARPORT proposed phase II/III randomized clinical trial, to conduct a survey of potential sites to determine the feasibility of the study, and to collaboratively develop the final study design and protocol to ensure robust enrollment and the successful completion of the study. Please refer to the attached VA STARPORT Executive Summary for more information about the trial.

Responses to items on the attached survey may be provided by your office, by section chiefs or by potential site investigator(s) for the study. If your Section Chief was not cc'd to this email, please forward this survey to him/her. Please coordinate your responses with all potential investigators and your prostate cancer multidisciplinary team.

We have also requested that your survey identifies potential study champions from medical oncology and urology as we believe that a multi-disciplinary research team is the key to the success of a study. We will rely on your guidance to identify the departments, sections and investigator(s) who are best qualified to represent your VA Medical Center.

We look forward to selecting VA sites and site investigators that can not only meet our enrollment targets but also be critical partners in designing the final study protocol, training and supervising site research staff and ensuring the overall successful implementation of the study.

We will contact you for further discussions. Please let me know if you have any additional questions about the trial. Thank you very much for your consideration of this study.

**Medical Center Information**

1. Medical Center Name:______________
2. Potential Lead Investigator Name and Position Title:____________________
3. Station Number:________________________
4. VISN Number :_________________________
5. Person(s)  completing survey:
   Name:__________________________________
   Position title:_____________________________
   E-mail:__________________________________
   Fax:____________________________________
6. Is your VA Medical Center interested in participating in VA STARPORT?

   ☐ Yes, **please complete survey below**
   ☐ No, please check all applicable reasons below:
   - ☐ Competing trials will preclude recruitment
   - ☐ Lack of Equipoise
   - ☐ Not enough patients eligible for the study
   - ☐ No available Site Investigator with sufficient time for or interest in the study
   - ☐ I am not interested now, but I would like to be contacted in the future
   - ☐ Other, please describe:

**A. Potential Patient Availability and Recruitment for VA STARPORT**? (Can be completed by the member of Service or Section likely to be responsible for conducting the study). Please review eligibility criteria from the attached study summary or draft protocol to answer following questions.

1. On average, approximately how many patients per month at your VAMC would be diagnosed with *de novo* or recurrent oligometastatic (1-10 nodal or distant metastases) prostate cancer on PSMA PET imaging after definitive therapy (excluding castration-resistant patients)?

   _______________

2. Considering the inclusion and exclusion criteria, approximately how many patients/month seen in clinics would likely be eligible for the study? _______________

3. Overall, please estimate what proportion of cases of oligometastatic prostate cancer at your station would be unable to participate due to distance travelled to the medical center and/or due to transfer to facilities or care providers outside of the VA? _______________

4. From what clinics/wards would patients be recruited for this study? Please indicate CBOCs and other referring VAMCs if you are a complex care referral site for other VAMCs.

   ______________________________________________________________________

   ______________________________________________________________________

5. What overall and specific strategies would you utilize to enhance potential subject contact, enrollment and retention in VA STARPORT?

   ______________________________________________________________________

   ______________________________________________________________________

5. After reviewing the proposed studies eligibility criteria, do you find any criteria that may be an issue with recruitment? Please specify:

   ______________________________________________________________________

   ______________________________________________________________________

6. Do you foresee any or other obstacles or challenges when implementing this protocol? If yes, please describe the issues. Do you have a proposed solution or an idea that might minimize the obstacle(s) or challenge(s)?

   ______________________________________________________________________

   ______________________________________________________________________

7. Are there any trials in the planning or submission phase that you will be participating in that could conflict with the conduct of STARPORT (e.g. through similar targeted subjects or your time commitment)?

   ______________________________________________________________________

   ______________________________________________________________________

**B. Investigator Availability**

1. What Clinical Service and Section at your VA Medical Center would most likely be responsible for carrying out this study?

   ______________________________

2. Name of Radiation Oncology Champion: ______________________________

3. Name of Urology Champion: ______________________________

4. Name of Medical Oncology Champion: ______________________________

5. Describe your clinical or research skills/experiences that position you well for successful participation as a local site investigator for VA STARPORT:

   ________________________________________________________________

   ________________________________________________________________

6. Describe your specific interest in or enthusiasm for participating in this trial:

   ________________________________________________________________

   ________________________________________________________________

7. On average how many hours per week can you dedicate to the proposed study:

   ____________

8. Do any of your current or recent clinical research experiences involve local therapy in patients with oligometastasis or SBRT? If yes, describe:

   ________________________________________________________________

   ________________________________________________________________

9. Given the information we supplied for the proposed study, do you or other members of your multidisciplinary team anticipate having:

   a. Financial conflict of interest? If yes, describe:

   ________________________________________________________________

   ________________________________________________________________

   b. Any concerns with clinical equipoise? If yes, describe:

   ________________________________________________________________

   ________________________________________________________________

10. If you are a relatively new investigator or with no experience in participating in randomized trials, is there a senior advisor or mentor in your section who can provide such supervision for you?
    No ____ N/A ____

**C. Research Staffing**

1. Is a research nurse or other type of study coordinator now at your VAMC who would likely be available to work on VA STARPORT at 0.5 FTE? Yes ____ No ____
   a. If Yes:
      i. When would that person be available to work on VA STARPORT? __________
      ii. How many years of clinical research experience does s/he have? __________
      iii. What degree and/or credentials (RN, PA, MD, PhD, CCRC, etc.) does s/he have? __________________
      iv. How many trials, if any, is the proposed study coordinator now engaged in and in what role and at what FTE? ________________________________________
   b. If you need to hire a study coordinator, what would be a realistic timeline for the hire?
2. Do you have a dedicated office area for your study staff (e.g. study coordinator) where study subjects can be interviewed and study records kept securely? Yes ____ No ____
3. Would such an office be convenient to patient care areas where study subjects would likely be seen clinically? Yes ____ No ____

**D. Diagnostic and Therapeutic Capabilities**

1. Do you have the ability to perform Fluciclovine or PSMA PET/CT for patients with prostate cancer? Yes ____ No ____
2. Do you routinely perform Fluciclovine or PSMA PET/CT for patients with *de novo* intermediate or high risk prostate cancer? Yes ____ No ____
3. Do you have the technical ability and expertise to deliver metastasis-directed SBRT at your VAMC? Yes ____ No ____
4. Do you have the ability and expertise to deliver salvage local therapy (i.e. salvage radiotherapy, radical prostatectomy, salvage brachytherapy) for patients with local failure as a component of oligorecurrence at your VAMC? Yes ____ No ____
5. Do you have the ability to access and upload data to an electronic data capture system? Yes ____ No ____
6. Do you have the ability to collect and transport biopsy materials and blood samples? Yes ____ No ____

**E. Other Comments (Use the space below to document any other comments pertaining to your site):**

______________________________________________________________________
______________________________________________________________________
______________________________________________________________________
______________________________________________________________________
